# Antiepileptic Drug Combinations for Epilepsy: Mechanisms, Clinical Strategies, and Future Prospects

**DOI:** 10.3390/ijms26094035

**Published:** 2025-04-24

**Authors:** Cunjiang Li, Xingyu Wang, Mingzhenlong Deng, Qinggen Luo, Chaoxing Yang, Zhicheng Gu, Shuxian Lin, Yongxiang Luo, Lei Chen, Yan Li, Bin He

**Affiliations:** 1 State Key Laboratory of Functions and Applications of Medicinal Plants, Engineering Research Center for the Development and Application of Ethnic Medicine and TCM (Ministry of Education), Guizhou Provincial Key Laboratory of Pharmaceutics, School of Pharmacy, Guizhou Medical University, Guiyang 550004, China; licunjiang7513@163.com (C.L.); wang1329086953@163.com (X.W.); dengmingzhenlong@163.com (M.D.); luoqinggen1017@163.com (Q.L.); yangchaoxing98@163.com (C.Y.); guzhicheng0520@163.com (Z.G.); linshuxian365@163.com (S.L.); luoyongxiang0616@163.com (Y.L.); leichen@gmc.edu.cn (L.C.); 2 School of Basic Medical Science, Guizhou Medical University, Guiyang 550004, China

**Keywords:** epilepsy, antiepileptic drugs (AEDs), combination therapy, precision medicine, drug synergy, multi-target drugs

## Abstract

Epilepsy is a chronic neurological disorder characterized by abnormal neuronal discharge, leading to recurrent and unpredictable disruptions in brain function. Despite over 30 antiepileptic drugs (AEDs), 30% of patients develop drug-resistant epilepsy, requiring combination therapy. This review explores epilepsy’s pathogenesis, including neuronal hyperexcitability, neurotransmitter imbalances, and ion channel dysfunction, alongside genetic, inflammatory, immune, and oxidative stress factors. AEDs are classified by mechanisms like voltage-gated ion channel modulation and GABA/glutamate regulation, tracing their evolution from traditional (e.g., phenobarbital) to modern therapies (e.g., lamotrigine). Combination therapy, using complementary mechanisms (e.g., lacosamide with levetiracetam), enhances efficacy but poses risks like drug interactions and cognitive impairment. Integrating molecular biology and pharmacology advances, this review highlights the need for rational drug selection and individualized strategies to improve epilepsy treatment outcomes and patient quality of life. Future directions include personalized treatments, optimized dosage forms, novel drug targets, and multi-target drugs.

## 1. Introduction

Epilepsy is a chronic neurological disorder characterized by abnormal neuronal discharge, leading to recurrent and unpredictable disruptions in brain function [1]. It ranks among the most common brain disorders globally, with an annual incidence rate ranging from 50.4 to 81.7 cases per 100,000 people [2]. Approximately 4.9 million new cases of epilepsy are diagnosed each year, contributing to a global burden of over 70 million people living with the condition [3,4]. The disorder manifests through a variety of symptoms, including convulsions, loss of consciousness, muscle rigidity, muscle relaxation, and prolonged muscle contractions, which can significantly impact an individual’s quality of life [5]. 

**Figure 1:**
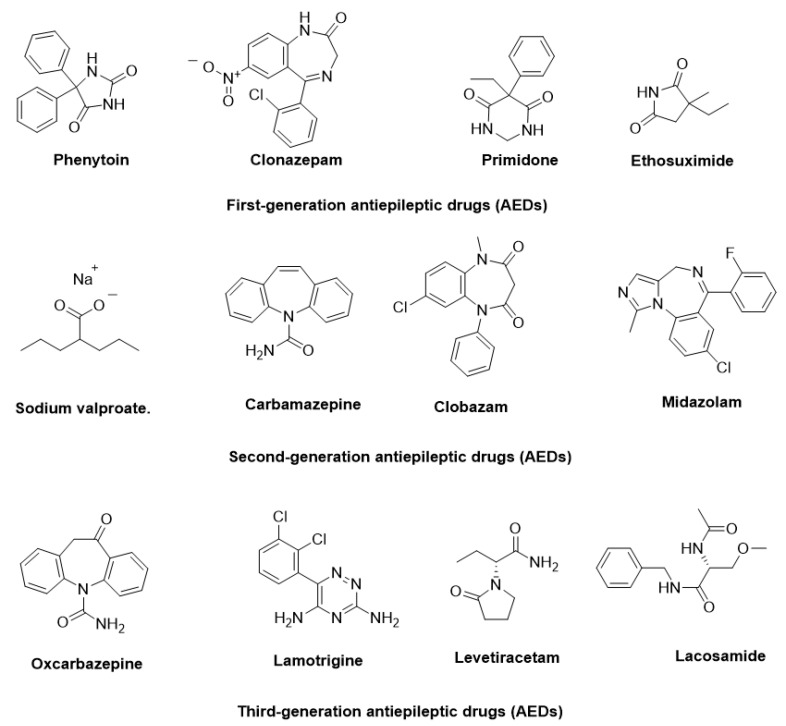
Representative antiepileptic drugs (AEDs).

The pathophysiology of epilepsy involves complex mechanisms, such as abnormal neuronal firing, neurotransmitter imbalances, ion channel dysfunction, and genetic predispositions. Additionally, factors like inflammation, oxidative stress, and immune responses further contribute to its onset and progression [6]. To manage epilepsy, more than 30 antiepileptic drugs (AEDs) have been approved for clinical use, targeting diverse mechanisms, including voltage-gated ion channel modulation, GABAergic transmission enhancement, and glutamate receptor inhibition [7]. In Figure 1, some AEDs that have been approved for the treatment of epilepsy are illustrated. Despite these advancements, approximately 30% of patients experience drug-resistant epilepsy, where monotherapy proves insufficient. For these patients, achieving therapeutic efficacy often requires polytherapy, involving the concurrent administration of two to four AEDs with complementary mechanisms of action, tailored to their specific condition.

Drug combination strategies are increasingly pivotal in epilepsy treatment, particularly for drug-resistant cases, as they offer synergistic effects to improve efficacy, reduce resistance, and minimize side effects. Personalized treatment regimens, which consider factors such as drug interactions, patient age, and individual genetic profiles, are essential for optimizing outcomes. However, the challenges of managing refractory epilepsy underscore the need for continued research into more effective therapies and a deeper understanding of the disorder’s underlying mechanisms. This comprehensive approach is vital for advancing epilepsy treatment and improving the quality of life for patients worldwide.

## 2. Pathogenesis of Epilepsy

### 2.1. Core Pathogenic Mechanisms in Epilepsy

**Figure 2:**
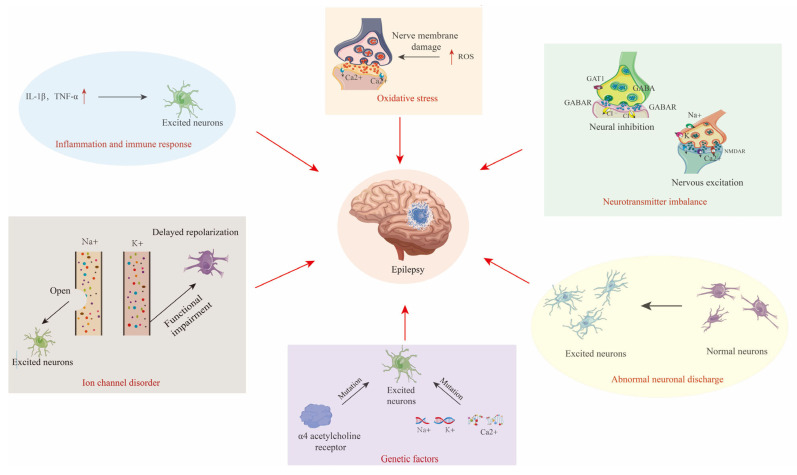
Diagram of the pathogenesis of epilepsy, including abnormal neuronal discharges, excitation–inhibition imbalance, and channelopathies, alongside synergistic interactions with genetic susceptibility, neuroinflammation, and oxidative stress. The red arrow indicates the causes of Epilepsy.

The core pathology of epilepsy revolves around the synergy of abnormal neuronal discharges, neurotransmitter imbalances, and ion channel dysfunction (Figure 2) [8,9,10,11,12].

Abnormal Neuronal Discharges and Synchronization

Epileptic activity originates from cortical “epileptic foci”, where neurons exhibit hyperexcitability and reduced inhibition due to genetic mutations, local inflammation, or metabolic disturbances, leading to synchronized discharges [9]. These discharges propagate to adjacent brain regions via synaptic connections or electrotonic spread, triggering widespread abnormal activity [10].

Bidirectional Regulation of Excitation–Inhibition Balance

The dysregulation of glutamate (an excitatory neurotransmitter) and γ-aminobutyric acid (GABA, an inhibitory neurotransmitter) is a hallmark of epilepsy [11]. Glutamatergic hyperactivity induces calcium ion (Ca^2+^) influx through N-methyl-D-aspartate receptors (NMDA receptors), causing excitotoxicity, while GABAergic interneuron loss or dysfunction exacerbates network hyperexcitability [12].

Molecular Basis of Channelopathies

Genetic defects in voltage-gated ion channels underlie multiple epilepsy syndromes. Sodium channels: mutations in SCN1A (voltage-gated sodium channel alpha 1 subunit) impair inhibitory interneuron function, which is strongly linked to Dravet syndrome [12]. Potassium channels: KCNQ2 (voltage-gated potassium channel subfamily Q member 2) mutations reduce M-currents, delaying neuronal repolarization in neonatal epilepsy [13].

### 2.2. Multifactorial Interaction Network in Epileptogenesis

Epileptogenesis is driven by dynamic interactions among genetic, inflammatory, and oxidative stress mechanisms (Figure 2) [14,15,16].

Epigenetic Modulation of Genetic Susceptibility

Beyond classical ion channel mutations, epigenetic modifications (e.g., DNA methylation, histone acetylation) regulate genes like SCN1A to modulate neuronal excitability [17,18]. For instance, the overexpression of microRNA-134 (miR-134) promotes dendritic spine aberrations via suppression of LIM kinase 1 (LIMK1) signaling, aggravating epileptic network remodeling [19].

Cascade Amplification of Neuroinflammation

Activated microglia and astrocytes release pro-inflammatory cytokines, such as interleukin-1β (IL-1β) and tumor necrosis factor-α (TNF-α), lowering seizure thresholds through blood–brain barrier disruption and peripheral immune cell infiltration [20], as well as the activation of the NOD-like receptor pyrin domain-containing 3 (NLRP3) inflammasome, inducing neuronal pyroptosis and synaptic loss [21,22].

Vicious Cycle of Oxidative Stress and Metabolic Dysfunction

Status epilepticus triggers mitochondrial dysfunction and reactive oxygen species (ROS) accumulation, damaging neuronal membranes and altering ion channel activity [23,24]. For example, defects in the nuclear factor erythroid 2-related factor 2 (Nrf2) antioxidant pathway exacerbate hippocampal oxidative damage, associated with drug-resistant epilepsy [25].

Mechanistic Synergy

Genetic backgrounds potentiate inflammatory responses (e.g., NLRP3 activation), while oxidative stress exacerbates electrophysiological abnormalities via ion channel modifications [26,27]. This multidimensional crosstalk constructs a complex network of epileptogenic mechanisms, offering novel targets for precision therapy.

## 3. Classification of Common Antiepileptic Drugs (AEDs)

### 3.1. Historical Development Stages of AEDs

Despite the complexity of epilepsy’s pathogenesis, its treatment methods are diverse. Pharmacotherapy, surgery, and ketogenic diets are classic approaches, with drug therapy being the primary treatment method due to its ability to effectively control seizures [15]. Based on current clinical practices and research, AEDs approved for clinical use can be broadly categorized into three historical development stages.

The first stage (late 19th century to mid-20th century) is characterized by the discovery of traditional drugs, including the introduction of phenytoin (1938) and ethosuximide (1958). These drugs effectively reduced seizures by controlling abnormal neuronal discharges, but they were associated with numerous side effects and drug interactions.

The second stage (mid-20th century) marked the emergence of various new AEDs, such as carbamazepine (1959) and valproic acid (1978), which offered broader efficacy for multiple types of epilepsy and fewer side effects, compared to the traditional drugs [16].

The third stage (1990s to present) is guided by precision medicine and individualized treatment. The development of new AEDs (e.g., lamotrigine (1994), lacosamide (2008), and levetiracetam (2009) focuses on targeted mechanisms, reduced adverse effects, and improved quality of life. This stage also involves in-depth research on drug interactions, providing a basis for personalized combination therapy [19]. These developmental stages have collectively advanced the diversity and scientific rigor in epilepsy treatment [17].

### 3.2. Classification of Common Antiepileptic Drugs

**Figure 3:**
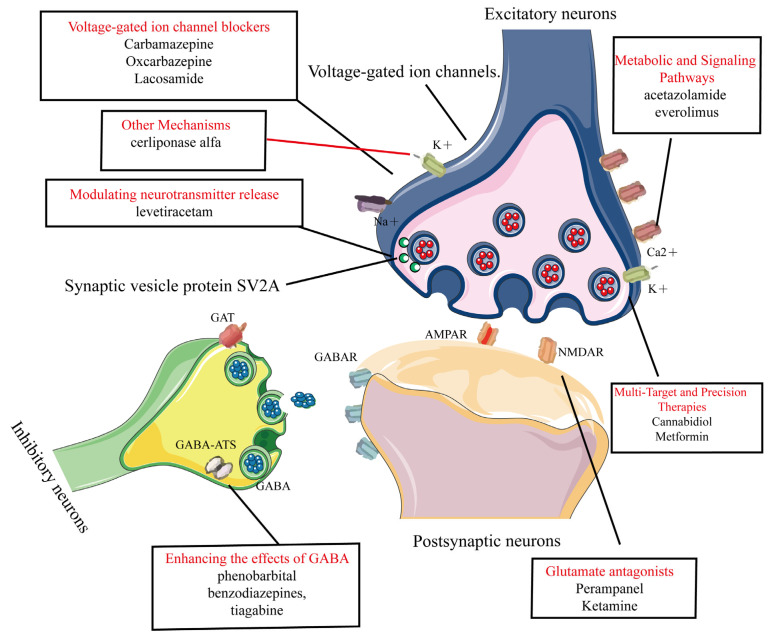
Common AEDs can be classified into six categories based on their mechanisms of action. This figure categorizes clinically used AEDs into seven mechanistic classes with representative agents, 1. Voltage-gated ion channel modulation. 2. Enhancement of GABA-mediated inhibitory neurotransmission. 3. Reduction of glutamate-mediated excitatory neurotransmission. 4. Modulation of presynaptic neurotransmitter release. 5. Novel agents targeting metabolic and signaling pathways. 6. Multi-target and precision therapies. 7. Other mechanisms.

Common AEDs can be classified into six categories based on their mechanisms of action, as shown in Figure 3:

1. Modulation of voltage-gated ion channels: This includes voltage-gated sodium, potassium, and calcium channels [20,28,29]. AEDs in this category include phenytoin, carbamazepine, oxcarbazepine, and lacosamide [21,22,23,24].
2. Enhancement of GABA-Mediated inhibitory neurotransmission: This involves agonizing GABA receptors (GABAR), inhibiting GABA transporters (GAT), and GABA transaminase (GABA-ATS). AEDs in this category include phenobarbital, benzodiazepines, and tiagabine [26,27,30].
3. Reduction of glutamate-mediated excitatory neurotransmission: This targets α-amino-3-hydroxy-5-methyl-4-isoxazolepropionic acid (AMPA) and N-methyl-D-aspartate (NMDA). Perampanel (AMPA receptor antagonist) is used for epilepsy but can cause dizziness, irritability, and psychiatric side effects [31,32]. Ketamine (NMDA receptor antagonist) is used for anesthesia and treatment-resistant depression, but has risks of abuse, dissociative effects, and cognitive concerns [33,34].
4. Modulation of presynaptic neurotransmitter release: This includes inhibiting synaptic vesicle protein 2A (SV2A) and alpha-2-delta protein (α2δ) proteins to reduce neuronal excitability. An example is levetiracetam [35,36,37], which works through this mechanism.
5. Novel agents targeting metabolic and signaling pathways: These include carbonic anhydrase (CA) inhibitors and Mammalian Target of Rapamycin (mTOR) inhibitors. Examples are acetazolamide and everolimus [38,39,40,41].
6. Multi-Target and Precision Therapies.
  Gene therapy: adeno-associated virus (AAV) vectors deliver the SCN1A gene in Dravet syndrome [42]. Metabolic–immune modulators: metformin improves mitochondrial function and suppresses NLRP3 inflammasome activation [43]. Transient receptor potential vanilloid 1 (TRPV1) modulators: cannabidiol (CBD) can enhance inhibitory microcircuits and acts via dual targeting of TRPV1/GPR55 [44].
7. Other mechanisms: these include inhibition of the Na^+^-K^+^-2Cl^−^ cotransporter 1 (NKCC1) transporter, modulation of neuronal growth and plasticity, and lysosomal enzyme replacement therapy (e.g., cerliponase alfa, which treats neuronal ceroid lipofuscinosis type 2 (CLN2) related epilepsy by supplementing deficient lysosomal enzymes) [45,46,47].

## 4. Combination Therapy Strategies

The use of AEDs is central to epilepsy treatment, and for patients who do not respond to monotherapy, rational combination therapy has become an important strategy in modern epilepsy management. This approach aims to enhance efficacy, reduce the development of drug resistance, and lower seizure frequency by combining drugs with different mechanisms of action. Combination therapy is not only applicable to common types of epilepsy, such as generalized and focal epilepsy, but also shows significant potential in managing refractory epilepsy. In recent years, with the development of new AEDs and advances in pharmacological research, the range of options for combination therapy has expanded. Additionally, through dynamic monitoring and dose adjustments tailored to individual patient needs, drug combinations can be further optimized to minimize treatment-related risks.

### 4.1. Basic Principles of Combination Therapy for Epilepsy

#### 4.1.1. Clinical Evidence

Pharmacotherapy remains the primary approach for epilepsy, and rational combination therapy has emerged as a critical strategy. In the ILAE guidelines, Glauser et al. systematically analyzed the efficacy of AEDs and proposed selecting drug combinations based on seizure types and syndrome characteristics, prioritizing complementary mechanisms to enhance therapeutic outcomes [48]. For example, combining lamotrigine with valproate improves efficacy, whereas phenytoin combined with topiramate may increase the risk of adverse effects [49]. Peng et al. demonstrated that different drug combinations vary in efficacy across epilepsy types: oxcarbazepine shows superior control of focal seizures, while valproate performs better in generalized seizures [50]. Kwan and Brodie found that although monotherapy achieves a success rate of approximately 50%, combination therapy significantly improves seizure control, though it requires careful, long-term monitoring of patient tolerance [51].

#### 4.1.2. Advantages

Further research has explored the principles of AED combinations, emphasizing the need to avoid combining drugs with overlapping mechanisms, while considering individualized factors, such as patient age and etiology, to reduce additive side effects [52]. Park et al. demonstrated that the effectiveness of drug combinations critically depends on drug interactions and the complementarity of their mechanisms [49]. Hachad found that newer AEDs (e.g., levetiracetam and lacosamide) exhibit lower interaction risks when combined with traditional drugs, offering both efficacy and safety advantages [53]. Sirmagul highlighted that optimized combinations (e.g., carbamazepine + valproate) can adjust plasma drug concentrations through enzyme induction or inhibition, thereby enhancing therapeutic outcomes [54].

Perucca and Gilliam proposed that combination therapy should avoid the additive effects of toxicity. The combination of topiramate and levetiracetam, for example, shows a good balance between efficacy and tolerability [55]. Perucca and Meador further explored the safety of newer AEDs, noting that their lower toxicity and fewer adverse effects make them preferable choices in combination therapy [56]. Kennedy and Lhatoo studied the central nervous system adverse effects of AEDs, emphasizing the need to pay special attention to cognitive and behavioral side effects in patients on long-term combination therapy [57]. Greenwood suggested avoiding the combination of highly toxic drugs [58]. Tatum conducted a comprehensive analysis of AED adverse effects and interactions, proposing that rational combination therapy design should focus on drug synergy while avoiding the additive adverse effects caused by interactions [59]. Regular monitoring of drug concentrations, dose adjustments, and dynamic optimization of treatment plans based on patient responses can maximize therapeutic efficacy and minimize side effects. Johannessen Landmark analyzed the impact of host factors on the pharmacokinetics of AEDs, highlighting that individual differences, such as age, gender, and genetic polymorphisms, play a key role in combination therapy [60]. Therefore, the design of individualized treatment plans should consider these factors to optimize drug effects. Ray et al. discovered that AI can be employed to identify responders to gabapentin treatment for alcohol use disorder [61].

#### 4.1.3. Considerations

Drug Interactions: Zaccara noted that certain AEDs (e.g., carbamazepine and valproate) may significantly alter the plasma concentrations of co-administered drugs through enzyme induction or inhibition, requiring careful consideration to avoid treatment failure or adverse effects [62]. Patsalos added that traditional AEDs (e.g., phenytoin and phenobarbital) may reduce the efficacy of other drugs, whereas newer AEDs (e.g., topiramate and lamotrigine) exhibit higher safety and compatibility in combination therapy [63]. Owing to potential interactions between antiepileptic drugs and cannabis, meticulous monitoring is essential to detect any aberrations in plasma levels [64].

Adverse Effect Management: Perucca and Gilliam proposed avoiding additive toxicity effects in combination therapy; for example, combining topiramate with levetiracetam balances efficacy and tolerability [55]. Perucca and Meador emphasized that newer AEDs, with their lower toxicity and fewer side effects, are preferable choices for combination therapy [56]. Kennedy and S.D. Lhatoo highlighted the need to monitor cognitive and behavioral side effects during long-term combination therapy [57], while Greenwood advised against combining highly toxic drugs [58]. Shakhatreh et al. have developed a deep learning model utilizing wearable devices, such as EEG headbands, and electronic health records data to predict epileptic seizures one hour in advance, with a sensitivity of 92%, and to monitor side effects in real-time, such as rashes and cognitive impairments, with an accuracy of 85%. This demonstrates the potential of AI in dynamically monitoring drug side effects [65].

Individualized Monitoring: Tatum suggested that regular monitoring of drug concentrations, dose adjustments, and dynamic optimization of treatment plans can maximize efficacy and minimize side effects [59]. Johannessen Landmark stressed that individual differences, such as age, gender, and genetic polymorphisms, play a critical role in combination therapy, necessitating tailored treatment plans [60].

**Figure 4:**
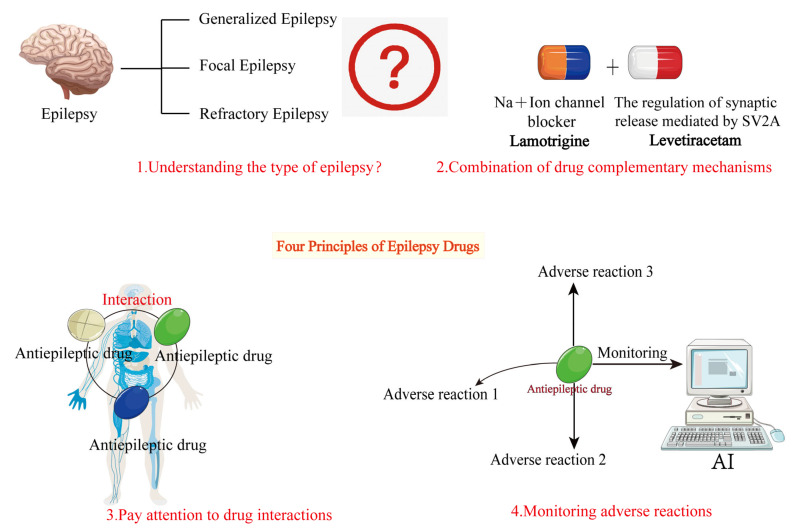
Basic principles of combination therapy for epilepsy. This figure outlines the fundamental principles of AED combination therapy. The basic principles are as follows: 1. Understand the type of epileptic seizures. 2. Choose antiepileptic drugs with complementary mechanisms. 3. Consider drug interactions when combining medications. 4. Monitor for adverse drug reactions during treatment.

The basic principles of combination therapy for epilepsy involve selecting drug combinations with complementary mechanisms based on a thorough understanding of seizure types, syndrome characteristics, and individual patient needs, while closely monitoring drug interactions and potential adverse effects. By rationally designing drug combinations, as shown in Figure 4, it is possible to enhance efficacy while minimizing treatment-related risks, providing safer and more effective treatment options for epilepsy patients.

### 4.2. Strategies for Antiepileptic Drug Combinations in Different Epilepsy Types

#### 4.2.1. Generalized Epilepsy

Generalized epilepsy is one of the most common types of epilepsy, encompassing various clinical manifestations, such as absence seizures and tonic–clonic seizures. The treatment of generalized epilepsy faces multiple challenges, including drug resistance, the diversity of seizure types, and long-term side effects. To improve treatment efficacy and reduce side effects, an increasing number of studies have focused on combination therapy strategies. Drug combinations not only enhance efficacy but also reduce the side effects of monotherapy through complementary mechanisms of action, thereby improving long-term patient outcomes.

In the treatment of generalized epilepsy, the combination of valproate and lamotrigine is often considered a first-line treatment option, as indicated in Table 1. The evaluation of the data in Table 1 assigns ratings based on either mechanistic complementarity or clinical evidence [66]. Valproate acts through multiple mechanisms, inhibiting neuronal hyperexcitability while enhancing inhibitory neurotransmission. Lamotrigine, on the other hand, stabilizes neuronal membrane depolarization to reduce abnormal neuronal discharges. The combination of these two drugs has demonstrated significant efficacy in numerous clinical studies, particularly in controlling tonic–clonic seizures. Research by Sarhan showed that the valproate–lamotrigine combination demonstrated a 75% reduction in tonic–clonic seizure frequency (*p* < 0.01) and was associated with a 60% lower risk of hepatotoxicity compared to traditional regimens [67].

The combination of levetiracetam and topiramate has shown advantages in controlling absence seizures. Levetiracetam reduces neuronal hyperexcitability by inhibiting synaptic vesicle release, while topiramate stabilizes neuronal electrical activity by modulating sodium channel activation [72,73]. Studies by Aneja and Sharma further validated the efficacy of this combination, particularly in reducing the frequency of absence seizures and minimizing side effects, outperforming traditional drug therapies [68].

With the emergence of new antiepileptic drugs, combination strategies have expanded. For example, the combination of lacosamide and levetiracetam has shown promising results, especially in patients with refractory epilepsy. Research by Mäkinen indicated that lacosamide and levetiracetam effectively reduce seizure frequency, and due to their pharmacokinetic properties, these new drugs can synergize with traditional drugs to optimize treatment outcomes [69]. This combination not only enhances efficacy but also reduces adverse effects, making it particularly suitable for patients resistant to traditional therapies.

The advantages of combination therapy extend beyond short-term efficacy to long-term management. Studies by Neyan demonstrated that rational drug combinations can effectively reduce relapse rates and improve patient adherence [74].

In addition to traditional antiepileptic drug combinations, ongoing drug development is advancing new treatment approaches. Löscher proposed that future antiepileptic drugs will increasingly focus on multi-mechanistic approaches to reduce seizures, with drug combinations significantly minimizing side effects. Löscher and Schmidt also emphasized that the optimization of drug combinations will greatly improve the treatment prospects for generalized epilepsy as new drugs continue to emerge [70,75]. It is worth noting that while new antiepileptic drugs offer additional options, traditional drugs like valproate remain important in treating generalized epilepsy. For instance, valproate continues to show high efficacy in managing absence and tonic–clonic seizures, despite its notable side effects. Research by Mattson indicated that adjusting doses and combining valproate with other drugs can better control seizures [76].

The treatment of generalized epilepsy is gradually shifting from monotherapy to combination therapy, which enhances efficacy, reduces side effects, and improves long-term outcomes. Drug selection is becoming more personalized, taking into account seizure types, drug resistance, and individual patient needs. As new drugs are developed and clinical research progresses, combination therapy strategies will play an increasingly important role in the treatment of generalized epilepsy, offering more possibilities and challenges.

#### 4.2.2. Focal Epilepsy

Focal epilepsy (also known as partial epilepsy) is one of the most common types of epilepsy, typically caused by excessive discharges in localized brain regions. While monotherapy can effectively control seizures in some patients, many, especially those with drug-resistant epilepsy, often require combination therapy to achieve better outcomes. The pharmacological treatment of focal epilepsy usually relies on the combined use of AEDs to enhance clinical efficacy. However, the selection and implementation of combination therapy must consider drug interactions, side effects, and individual patient differences.

Combination therapy for focal epilepsy is often based on complementary mechanisms of action to enhance treatment efficacy. For example, valproate and carbamazepine (or their derivatives, like oxcarbazepine) are frequently combined because they act through different mechanisms: valproate primarily increases GABA activity and inhibits sodium channels to control seizures, while carbamazepine stabilizes cell membranes and inhibits abnormal sodium channel discharges. They can achieve a 65% response rate, with a manageable risk of hyponatremia (incidence rate of 10%) [63]. Therefore, drug concentrations must be closely monitored during combination therapy to ensure they remain within the therapeutic range.

Although combination therapy often improves efficacy in focal epilepsy, it may also introduce certain side effects. For patients on long-term treatment, drug side effects cannot be overlooked. Research by Park and Kwo showed that traditional AEDs like carbamazepine and phenytoin commonly cause side effects such as drowsiness, dizziness, and cognitive impairment [77]. These side effects can impact patients’ quality of life, particularly for those requiring long-term medication, making side effects a significant barrier to treatment. Thus, when selecting drug combinations, it is essential to balance efficacy and side effects to avoid excessive adverse effects.

Medication adherence remains a critical issue in antiepileptic drug therapy, particularly in the long-term treatment of focal epilepsy. Factors such as side effects, dosing complexity, and drug costs can affect adherence. Buck identified these as key factors influencing patient adherence [78]. In the context of combination therapy for focal epilepsy, clinicians must optimize drug regimens to reduce side effects and simplify treatment plans to improve adherence. Strategies such as using fewer drugs with lower side effects or long-acting formulations can enhance adherence.

Drug interactions are a significant concern in combination therapy, especially for focal epilepsy. Vecht noted that certain drugs may alter the metabolism of others by affecting liver enzyme systems, impacting their efficacy and side effects [79]. For example, valproate, a potent CYP450 enzyme inhibitor, may slow the metabolism of other drugs, increasing their plasma concentrations. Therefore, clinicians must thoroughly understand drug interactions when designing combination therapies to avoid unstable efficacy or increased side effects. Thus, clinicians must evaluate patients’ overall medication profiles to ensure drug interactions do not compromise seizure control. Therefore, the treatment of focal epilepsy should adopt an individualized approach, selecting appropriate drug combinations based on patient-specific factors. For example, elderly patients with slower drug metabolism may require dose adjustments to avoid drug accumulation, while younger patients with better tolerance may benefit from more potent drug combinations.

With advances in antiepileptic drug research, the development of new drugs has expanded treatment options for focal epilepsy. Das et al. investigated FDA-approved drug combinations, suggesting that structurally diverse combinations may yield better outcomes [80]. These new drugs often have improved pharmacokinetic properties and act through more precise mechanisms, reducing side effects and enhancing efficacy in treating focal epilepsy. Leach et al. emphasized that the safety of AEDs cannot be overlooked, particularly for patients on long-term treatment, as side effects may have lasting impacts [81]. Research recommends careful drug selection and monitoring to ensure treatment safety Dalkara and Karakurt noted significant progress in AED development, with new drugs offering better seizure control and optimized safety profiles, providing more options for focal epilepsy patients [82].

In conclusion, the treatment of focal epilepsy requires selecting the most appropriate drug combinations based on individual patient characteristics. Rational drug combinations can enhance efficacy, reduce side effects, and improve quality of life. However, drug interactions may affect efficacy and side effects, necessitating close monitoring of treatment responses and drug concentrations. With the emergence of new drugs, the prospects for combination therapy are promising, but individualized treatment strategies and ongoing clinical observation are essential to optimize outcomes.

#### 4.2.3. Refractory Epilepsy

Refractory epilepsy refers to a type of epilepsy that remains uncontrolled, despite adequate treatment with AEDs. Its definition and diagnostic criteria are crucial in clinical practice. Breemen et al. suggested that complex etiologies and seizure patterns are significant factors contributing to refractory epilepsy, necessitating multidisciplinary collaboration and rational AED combinations [83]. Walia et al., through a systematic review of AED tolerability and safety, proposed prioritizing drug combinations with fewer side effects and better patient tolerance in refractory epilepsy treatment [84]. Anderson et al. further analyzed the treatment characteristics of elderly patients with refractory epilepsy, highlighting the need to consider pharmacokinetic changes due to reduced drug metabolism in this population to avoid drug accumulation and exacerbated side effects [85]. In the development and application of newer AEDs, Perucca and Meador emphasized the significant role of new drugs in improving the quality of life for patients with refractory epilepsy [71]. They also noted that certain drug combinations, such as lamotrigine and levetiracetam, offer complementary mechanisms that enhance seizure control and reduce cognitive side effects.

Madsen et al. investigated the potential of GABA transporters as targets for AEDs, suggesting that newer drugs can significantly enhance efficacy when combined with traditional AEDs [86]. Borowicz et al. found through animal studies that melatonin can enhance the protective effects of traditional AEDs (e.g., phenytoin), providing experimental evidence for combining newer and traditional drugs [87]. Benassi et al. summarized advancements in AED research over the past two decades, highlighting the breakthrough significance of newer drugs in treating refractory epilepsy and noting that their combination with existing drugs can significantly improve seizure control [88].

In the adjustment and optimization of polytherapy regimens, Ashli et al. studied the interactions of different drug combinations in clinical practice and their impact on efficacy, emphasizing the importance of drug selection and dose adjustments in optimizing treatment [89]. Mula and Sander explored the negative emotional impacts of AEDs, recommending prioritizing drugs with minimal emotional effects in polytherapy [90]. Refractory epilepsy is often associated with structural brain abnormalities, such as hippocampal sclerosis or cortical malformations [91]. These changes can create hyperexcitable neuronal networks, making it difficult to control seizures with standard AEDs. Corrales-Hernández et al. reviewed the history of AED development, emphasizing the need to consider individualized treatment needs and drug interaction profiles in combination strategies to ensure efficacy and long-term patient adherence [92].

### 4.3. Potential Risks of Combination Therapy

Combination therapy with AEDs plays a significant role in improving seizure control and quality of life, particularly in patients with refractory epilepsy and complex seizure types. However, this strategy also carries potential risks, including adverse effects from drug interactions, reduced patient adherence, impaired cognitive and psychological function, and specific impacts on special populations (e.g., adolescents, elderly patients, and those with comorbidities). Systematic studies in the literature provide important insights for clinical practice.

Moreira-Silva et al. noted that AED combination therapy may disrupt neurotransmitter balance in the central nervous system, leading to cognitive dysfunction, particularly with drug combinations affecting the GABA system [93]. Patients may experience attention deficits, memory impairment, and other cognitive issues, which can worsen with higher doses and have long-term impacts on social functioning and quality of life. The study also found that certain drug combinations may amplify adverse effects when used with cognitive stimulants (e.g., caffeine or cannabinoids), necessitating dynamic cognitive assessments during treatment. Brookles et al. highlighted the potential impact of AEDs on cardiac rhythm, especially in epilepsy patients with underlying cardiovascular conditions. Some AED combinations (e.g., levetiracetam with carbamazepine, or valproate with topiramate) may cause arrhythmias or exacerbate existing heart issues, particularly with high doses or in patients with undiagnosed heart conditions [94]. This underscores the need for close cardiac monitoring during treatment to avoid serious adverse events.

Donzelli C further elaborated on the unique risks faced by adolescent patients in AED combination therapy [95]. Adolescents are in a rapid developmental stage, and long-term AED use may increase the risk of drug dependence or tolerance, particularly with benzodiazepines. This dependence can negatively impact mental health and complicate treatment. The study also found that long-term drug dependence in adolescents may reduce treatment adherence, affecting the outcomes.

Odhiambo et al. studied the association between polytherapy and psychiatric comorbidities in epilepsy patients, finding that long-term polytherapy may significantly increase the risk of depression, anxiety, and other mental health disorders [96]. This risk is closely related to the complexity of drug combinations, individual psychological states, and dose adjustments, suggesting the need for psychological interventions to reduce psychiatric comorbidities. Löscher focused on the impact of drug combinations on neuroplasticity, noting that some combinations may induce neuroadaptive changes through long-term modulation of neuronal excitability, negatively affecting neurofunctional recovery [97]. This phenomenon is particularly pronounced in children and adolescents, potentially leading to reduced learning ability or increased behavioral issues. Mangiardi et al. analyzed the combined use of AEDs and novel oral anticoagulants (NOACs) in post-stroke epilepsy patients, showing that this combination significantly improves seizure control but may also increase bleeding risk [98]. This risk is particularly concerning in elderly patients due to impaired coagulation and reduced liver and kidney function, highlighting the need for regular monitoring of hematological and renal parameters. Dasgupta et al. discussed the treatment risks in pediatric patients, finding that certain AEDs may exacerbate dizziness or motor coordination issues by affecting the vestibular system, negatively impacting daily life and social activities [99]. This underscores the need to balance treatment benefits with potential developmental impacts in children.

Lai et al. suggested that certain AED combinations may increase fracture risk by affecting bone metabolism, particularly in female patients [100]. Long-term use of drugs like valproate may reduce bone density, especially without adequate calcium and vitamin D supplementation. This bone metabolism abnormality may not be apparent early in treatment but can significantly increase skeletal-related adverse events over time. Cortes-flores et al. focused on patients with both epilepsy and dementia, finding that polytherapy may worsen cognitive decline and psychiatric symptoms [101]. This highlights the need to prioritize drug combinations with minimal cognitive impact in patients with neurodegenerative diseases and to regularly assess cognitive function.

While AED combination therapy offers significant therapeutic benefits, it also carries complex potential risks. These risks include drug interactions, adverse effects, long-term safety concerns, and multifaceted impacts on cognition, mood, and adherence.

### 4.4. Future Research Directions and Development Trends

#### 4.4.1. Personalized Treatment Plans Based on Molecular Mechanisms

The future of AED combination therapy lies in deciphering molecular mechanisms and designing personalized treatment plans. By investigating the molecular and genetic mechanisms of AEDs, researchers aim to optimize drug combinations for enhanced efficacy and reduced adverse effects [102]. Carmen R et al. revealed neurotoxic mechanisms triggered by AEDs, where certain drugs disrupt intracellular ion channels and oxidative stress pathways, leading to neuronal damage [103]. This insight offers a novel perspective for selecting drug combinations that minimize molecular toxicity. Husna and Kurniawan further explored the biomolecular roles of GABA and glutamate systems in epilepsy, proposing that understanding drug–pathway interactions could guide personalized drug selection and dosing adjustments for precise treatment [104].

Abaza et al. investigated non-traditional applications of AEDs, such as valproate’s dual role as an antiepileptic and histone deacetylase inhibitor [105]. Their study on valproate–proteasome inhibitor combinations demonstrated antiproliferative and pro-apoptotic effects in cancer cells. Although focused on oncology, these findings provide valuable insights for designing AED combinations with multi-mechanistic synergy. Serrano-Castro et al. applied personalized medicine to autoimmune-related epilepsy, showing that cenobamate–clobazam combinations significantly improved outcomes in anti-GAD65 antibody-associated cases [106]. This highlights the potential of molecular and immune-based strategies in complex epilepsy. Y. Liu et al. emphasized precision nutrition approaches like the ketogenic diet as promising adjuncts for epilepsy management [107].

Giannotta et al. advocated integrating patients’ biological rhythms into treatment plans [108]. Their research on pediatric neurological disorders suggests that synchronizing drug administration with physiological rhythms could maximize therapeutic benefits. Collectively, these studies underscore the transformative potential of combining molecular insights with personalized strategies, advancing AED development toward precision medicine.

#### 4.4.2. Reducing Risks in Combination Therapy Through Dosage Form Optimization

Combination therapy with AEDs is critical for improving seizure control, but its complexity raises risks of drug interactions and adverse effects. Optimizing dosage forms has thus emerged as a key strategy to mitigate these risks. Simplified regimens and advanced formulations can enhance adherence and reduce side effects. Kwan and Brodie highlighted that complex dosing regimens often lead to poor adherence, while drug interactions exacerbate risks [109]. They proposed fixed-dose combinations (FDCs)—single tablets containing multiple drugs—to reduce pill burden and dosing errors, particularly benefiting patients with refractory epilepsy requiring long-term polytherapy.

Perucca et al. emphasized the direct correlation between adverse effects and drug dosage [110]. High doses or inappropriate combinations may cause CNS dysfunction or organ damage. They recommended low-dose polytherapy combined with extended-release formulations to stabilize plasma concentrations and minimize fluctuations, thereby maintaining efficacy while reducing toxicity. This approach is especially vital for pediatric and elderly patients who are sensitive to pharmacokinetic variations.

Mula M and Sander J W underscored the role of long-acting formulations in optimizing AED therapy [90]. These formulations stabilize drug levels, reduce dosing frequency, and minimize errors. For chronic epilepsy patients, extended-release forms mitigate concentration fluctuations and adverse effects, particularly improving adherence in vulnerable populations like children and the elderly. Perucca et al. further noted that innovative dosage forms, such as transdermal patches or nanoparticle-based delivery systems, can bypass hepatic metabolism and reduce enzyme-related interactions [111]. Nanotechnology-enabled targeted delivery systems precisely release drugs at lesion sites, minimizing systemic toxicity and offering groundbreaking solutions for future AED combinations.

Optimizing dosage forms to reduce the risks of combination therapy is a critical direction in future epilepsy treatment. The development of FDCs, extended-release formulations, and long-acting dosage forms can minimize drug interactions and adverse effects while improving adherence and efficacy. Innovations in drug delivery systems and administration routes provide additional solutions for AED combination therapy. These advancements, combined with rational drug selection and personalized treatment strategies, will help patients achieve safer and more effective treatment outcomes. Through dosage form optimization and dose management, AED combination therapy is moving toward precision and personalization, offering epilepsy patients a higher quality of life and better long-term prognosis.

#### 4.4.3. New Combination Strategies and Novel Targets

Exploring new drug combinations and therapeutic targets is pivotal for advancing AED combination therapy. By leveraging multi-mechanistic synergy, researchers aim to enhance efficacy while mitigating risks. Li Y et al. investigated the pharmacological components and molecular mechanisms of gastrodin in hypertension, offering insights for network pharmacology-driven drug development [112]. Their study demonstrated that molecular docking and absorption analysis could uncover novel therapeutic potentials in existing drugs, enabling precise AED combinations with minimized off-target interactions. Alothman et al. explored the combined use of etanercept and pregabalin in a neuropathic pain model, showing synergistic reduction in hyperalgesia through distinct mechanisms [113]. Although focused on pain, these findings illuminate pathways for optimizing AED combinations via neural pathway modulation. Walker et al. highlighted drug repurposing for status epilepticus, emphasizing the potential of non-traditional AEDs to target novel neural network nodes and rebalance excitation–inhibition [114]. This strategy accelerates therapeutic discovery while addressing long-term safety concerns.

Martinez Elj studied excitation–inhibition balance at the neural network level in neurodevelopmental disorders, underscoring its centrality in epilepsy [115]. They proposed neurophysiological approaches to optimize AED combinations by modulating specific neural circuits, offering new targets for refractory epilepsy. Palmieri et al. integrated advances in neuroanatomy, suggesting that analyzing functional connectivity and structural changes in epilepsy-related brain regions could guide precise drug targeting [116]. Combining region-specific AEDs may locally regulate abnormal activity, improving efficacy while reducing systemic side effects.

Therefore, exploring new drug combinations and therapeutic targets is driving AED research toward more effective and safer treatments. From molecular mechanisms to neural network regulation and anatomical precision, these studies provide critical support in optimizing existing drug combinations and discovering new targets. These advancements not only enhance epilepsy treatment efficacy but also significantly reduce the risks of combination therapy, offering patients more personalized and precise treatment options.

#### 4.4.4. Development of Dual- and Multi-Target Drugs

The development of multi-target drugs represents a transformative trend in AED therapy. By acting on multiple pathological nodes within a single molecule, these drugs reduce the complexity and risks of polytherapy while enhancing efficacy. Löscher systematically compared single-target, multi-target, and polytherapy approaches, highlighting the potential of multi-target drugs to modulate excitation–inhibition balance in neural networks [117]. Such drugs improve overall efficacy by targeting critical pathways, circumventing drug interactions, and adverse effects common in traditional combinations. Löscher and Klein proposed novel methodologies for multi-target drug design, emphasizing the need to integrate seizure control, disease modification, and preventive potential. By targeting neuroinflammation, oxidative stress, and ion channel dysfunction, these drugs address multiple pathological mechanisms simultaneously, offering innovative solutions for refractory epilepsy and related neurodegenerative disorders [118].

Strac et al. emphasized the role of monoaminergic mechanisms in epilepsy, suggesting that multi-target drugs balancing monoaminergic activity could improve seizure control while mitigating cognitive and emotional impairments [119]. Kumari et al. reviewed the progress of multi-target AEDs, noting that existing drugs already exhibit multi-mechanistic properties (e.g., dual modulation of sodium channels, calcium channels, and GABAergic systems) [120]. Their high efficacy and low toxicity profiles provide a blueprint for future drug development. Talevi argued that traditional single-target strategies are increasingly limited, advocating for multidisciplinary collaboration in pharmacology, neuroscience, and computational biology to advance multi-target drug discovery [121].

**Figure 5:**
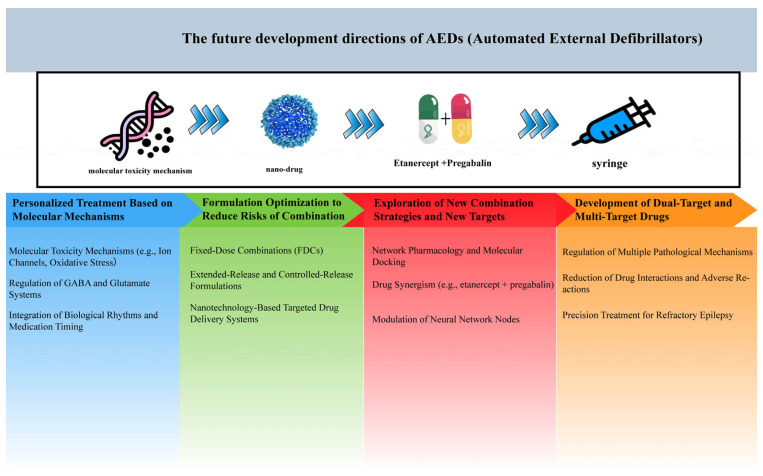
Future directions for AEDs development. This figure outlines the evolution of antiepileptic drugs from traditional single-target approaches to innovative multi-target therapies.

Clinically, French et al. demonstrated the promise of XEN1101, a novel multi-target drug that opens KCNQ potassium channels and inhibits glutamate release [122]. In focal epilepsy patients, XEN1101 achieved a 50% responder rate (vs. 28% placebo) with no serious cardiovascular side effects, validating the clinical viability of multi-target strategies. The development trends of AEDs are illustrated in Figure 5.

In conclusion, the development of dual- and multi-target drugs holds significant theoretical and practical value in epilepsy treatment. By targeting multiple pathological mechanisms within a single molecule, these medications boast the advantages of a broad therapeutic window and manageable safety profile, while also reducing costs and enhancing patient experience. This approach represents a promising direction for future AED research and therapy.

## Figures and Tables

**Table 1:** Commonly used drug combinations in clinical practice.

Types of Epilepsy	Combinations of Different Mechanisms	Reduced Drug Interactions	Reduced Adverse Drug Reactions
Generalized Epilepsy	Lacosamide + Sodium Valproate *** [67]	Levetiracetam + Topiramate **** [68]	Lacosamide + Levetiracetam *** [69]
Focal Epilepsy	Carbamazepine + Sodium Valproate ** [70]	-	-
Refractory Epilepsy	Lamotrigine+ Levetiracetam **** [71]	-	-

Symbol Key: ** (High Risk); *** (Theoretically Sound); **** (Strong Synergy with Preclinical Evidence).

## Data Availability

No data were used for the research described in the article.
